# Underwater sound production varies within not between species in sympatric newts

**DOI:** 10.7717/peerj.6649

**Published:** 2019-03-28

**Authors:** Jiří Hubáček, Monika Šugerková, Lumír Gvoždík

**Affiliations:** 1 Department of Botany and Zoology, Masaryk University, Brno, Czech Republic; 2 Institute of Vertebrate Biology of the Czech Academy of Sciences, Brno, Czech Republic

**Keywords:** Acoustic interference, Species recognition, Amphibians, Individual variation, Salamander, Sound production, Acoustic divergence

## Abstract

Sound production is a widespread phenomenon among animals. Effective sound use for mate or species recognition requires some acoustic differentiation at an individual or species level. Several species of caudate amphibians produce underwater sounds, but information about intra- and interspecific variation in their acoustic production is missing. We examined individual, sex, and species variation in underwater sound production in adults of two sympatric newt taxa, *Ichthyosaura alpestris* and *Lissotriton vulgaris*. Individual newts produced simple low- (peak frequency = 7–8 kHz) and mid-high frequency (14–17 kHz) clicks, which greatly overlap between sexes and species. Individual differences explained about 40–50% of total variation in sound parameters. These results provide foundations for further studies on the mechanisms and eco-evolutionary consequences of underwater acoustics in newts.

## Introduction

Sound production is a common attribute of diverse animal taxa. Their sounds mediate information about mates, food, or predators over long distances without the necessity of interaction with its producer (Wilkins, Seddon & Safran, 2013). In other taxa, sound production allows effective spatial orientation (Griffin, 1944; Siemers et al., 2009; Brinkløv, Fenton & Ratcliffe, 2013). However, various biotic and abiotic environmental factors interfere with specific sound transmissions. Accordingly, the combination of adaptive, i.e., ecological and sexual selection, and neutral, i.e., random drift, evolutionary processes (Wilkins, Seddon & Safran, 2013) gave rise to an immense diversity of produced sounds. While con- and heterospecific acoustic variation has received ample attention in some groups, such as insects, anurans, birds, bats, or primates, it has remained largely unexplored in other taxa.

Caudate amphibians, salamanders and newts, are animal group, in which sound production has received little attention. In comparison with the well-studied acoustic communication in anurans, available information about their sound production is limited to a few taxa (Maslin, 1950; Wells, 2007). Terrestrial salamanders produce low intensity sounds, such as hisses, clicks, or squeaks, when threatened, during mating or agonistic encounters. Some species produce underwater clicks, squeaks, or hissing sounds (Gehlbach & Walker, 1970; Wyman & Thrall, 1972; Davis & Brattstrom, 1975; Crovo, Zeyl & Johnston, 2016), for which the purpose is unknown. It may play a role in social interactions (Gehlbach & Walker, 1970; Davis & Brattstrom, 1975), in echolocation (Gehlbach & Walker, 1970), or are unintentional (Maslin, 1950). Although caudate amphibians lack an ear opening and middle ear, this poses no limitation for their high frequency hearing underwater. Their ability to detect sounds covers wide frequency ranges (Bulog & Schlegel, 2000), whereas others reported declining underwater hearing abilities towards high frequencies (Crovo, Zeyl & Johnston, 2016; Zeyl & Johnston, 2016). Despite mixed information about the extent of underwater hearing in salamanders, their conspecific sound detection ability may depend not only on hearing capabilities but also on acoustic divergence between individuals, sexes, or species occupying the same habitat, similar to anurans (Littlejohn, 1977; Gerhardt & Schwartz, 1995; Leininger & Kelley, 2015; but see Amézquita et al., 2011). Unfortunately, information about individual, sex, and species variation of underwater sound production is missing in this group.

In this study, we examined intra- and interspecific variation of underwater sound production in two European newts, *Ichthyosaura alpestris* (Laurenti, 1768) and *Lissotriton vulgaris* (Linnaeus, 1758). Both species are widely distributed across Europe with highly overlapping ranges (Roček, Joly & Grossenbacher, 2003; Schmidtler & Franzen, 2004). In addition, they frequently occur in the same water bodies (Van Buskirk, 2007). Aquatic newts are largely crepuscular or nocturnal, and water in their habitats is often turbid. Hence, newt space orientation, or mate and species recognition could be mediated by something other than visual cues. Accordingly, we predicted that underwater acoustic production is more widespread in newts than available information shows. If these sounds contribute to newt mate or species recognition, they should vary between sexes and (or) sympatric species, respectively.

## Materials & Methods

### Study species

**Figure 1:**
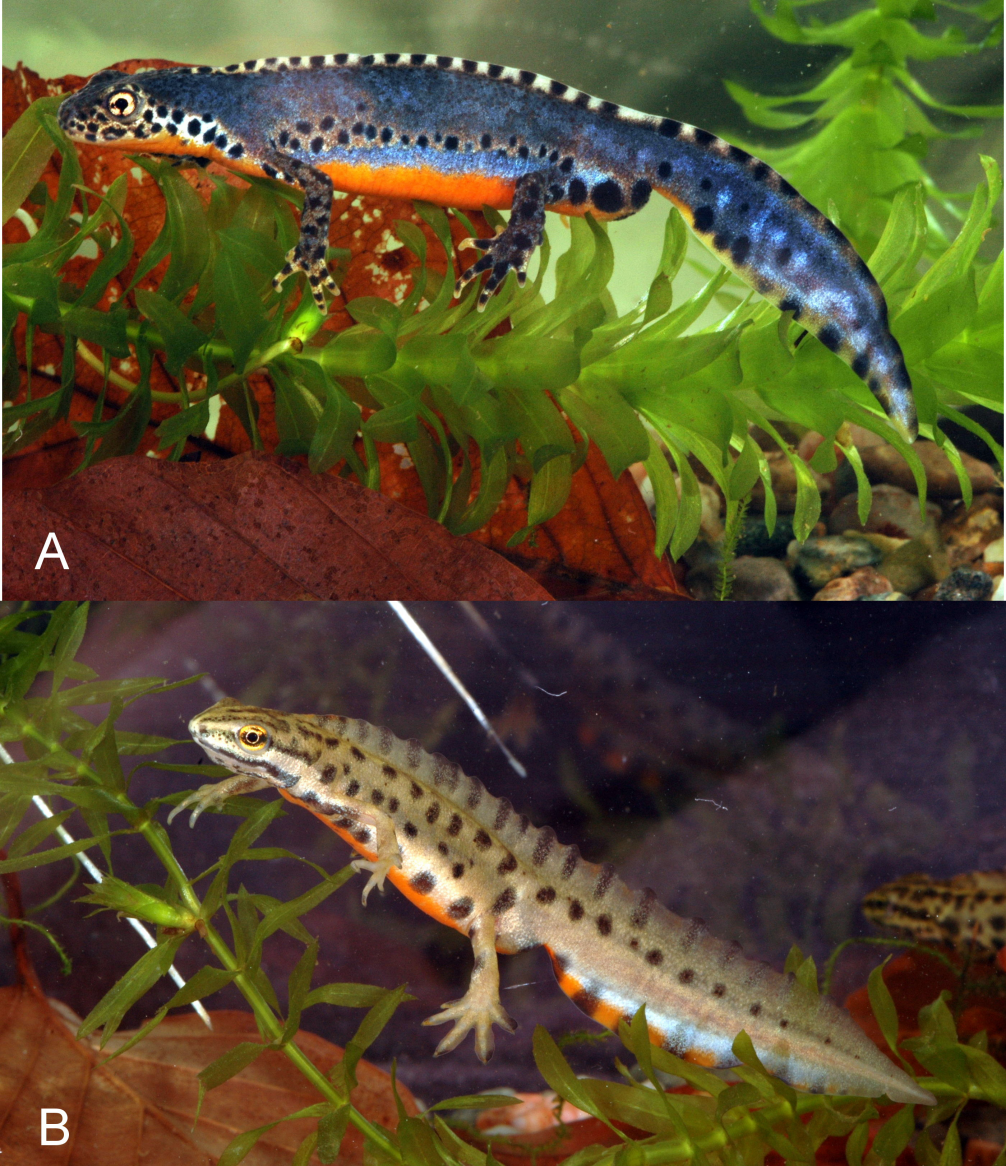
Study species. (A) *Ichthyosaura alpestris*, (B) *Lissotriton vulgaris*. Both specimens are males as seen during their aquatic phase.

*Ichthyosaura alpestris* and *L. vulgaris* are small to medium-sized (total length = 10–12 cm) newts (Fig. 1). They usually have a biphasic, i.e., aquatic and terrestrial, lifestyle. Their aquatic reproductive period lasts from April until June in Europe. They occur in various water bodies, from temporary pools to lakes. Concerning their acoustic repertoire, these newts occasionally produce squeaks when handled or clucking sounds whilst gulping air at the water surface (Maslin, 1950).

For this study, we captured adult *I. alpestris* (*n* = 17; body mass [BM] = 3.07 ± 0.95 [SD] g) and *L. vulgaris* (*n* = 13; BM = 1.65 ± 0.30 g; see Table 1 for sex ratio) from two pools (aerial distance 4 km) near Jihlava, Czech Republic. After transport to the research facility, groups of newts (*n* = 6–9) were placed into tanks (90 × 63 × 47 cm) located outdoors in semi-shade. Previous studies showed that these conditions provide similar light and temperature conditions as in the native habitat of newts (Smolinský & Gvoždík, 2013). Each tank was equipped with a piece of styrofoam to allow newts to leave the water. Some aquatic weeds (*Egeria densa*) and dead beech leaves were added to the bottom to provide hiding places. Food including live chiromid larvae, *Tubifex* worms and animal plankton, was provided *ad libitum*. Newts were left undisturbed under these conditions for at least three days prior to sound recording trials. All experimental procedures were approved by the Expert Committee for Animal Conservation of the Institute of Vertebrate Biology AS CR (research protocol no. 135/2016). The Agency for Nature Conservation and Landscape Protection of the Czech Republic issued permission to capture the newts (KUJI 224/2013).

**Table 1:** Descriptive statistics of underwater sounds. Parameters (mean ± SD, minimum, maximum) of underwater sound production in two newt species.

Parameters	*Ichthyosaura alpestris*		*Lissotriton vulgaris*	
	Males *N* = 6	Females *N* = 11	Males *N* = 6	Females *N* = 7
Low clicks	*n*= 155	*n*= 319	*n*= 150	*n*= 181
Duration (ms)	7.67 ± 2.82 2.82, 15.59	10.74 ± 4.15 2.90, 26.10	8.88 ± 2.75 2.80, 24.60	7.97 ± 3.85 2.13, 21.42
Interval (s)	4.72 ± 8.53 0.02, 47.28	0.84 ± 1.80 0.02, 18.77	1.55 ± 3.20 0.01, 19.65	1.08 ± 1.72 0.01, 9.03
Low frequency (kHz)	5.38 ± 1.50 1.50, 8.49	4.92 ± 1.22 0.91, 8.87	5.57 ± 1.01 0.46, 6.93	5.12 ± 1.07 1.07, 7.85
High frequency (kHz)	14.65 ± 4.44 4.44, 23.84	18.29 ± 4.83 4.83, 26.13	12.19 ± 4.12 4.12, 24.09	14.61 ± 5.84 5.84, 23.93
Peak frequency (kHz)	7.93 ± 1.23 1.23, 10.69	7.81 ± 0.78 0.78, 10.88	7.63 ± 0.47 0.47, 9.75	7.46 ± 0.78 0.78, 10.69
Mid-high clicks	*n*= 128	*n*= 229	*n*= 150	*n*= 169
Duration (ms)	11.53 ± 3.97 3.50, 22.10	11.93 ± 4.22 4.20, 26.10	10.60 ± 3.12 3.10, 21.90	12.14 ± 4.33 3.50, 28.10
Interval (s)	7.20 ± 12.20 0.01, 73.29	2.21 ± 3.91 0.02, 24.00	2.68 ± 4.31 0.01, 23.21	2.94 ± 5.71 0.01, 45.42
Low frequency (kHz)	7.21 ± 3.33 1.55, 17.16	8.22 ± 3.56 2.83, 17.60	8.02 ± 3.27 2.06, 18.43	10.06 ± 4.03 3.14, 17.82
High frequency (kHz)	21.89 ± 2.70 2.70, 26.94	22.15 ± 2.47 2.47, 26.94	22.48 ± 1.89 1.89, 25.16	22.37 ± 2.12 2.12, 26.39
Peak frequency (kHz)	15.35 ± 2.29 2.29, 20.06	15.80 ± 2.59 2.59, 21.00	14.62 ± 1.57 1.57, 20.44	16.48 ± 2.74 2.74, 20.81
Total click number	615 ± 573 36, 1875	2,579 ± 3,094 49, 8298	2,783 ± 1,599 9, 1056	3,968 ± 7,650 49, 8298

**Notes.**

*N* number of individuals *n* number of samples

### Sound recording and analysis

Newt sounds were recorded in eight plastic tanks (50 × 30 × 18 cm) placed in a walk-in environmental chamber at 15 °C. Each tank was filled with 15 l of non-chlorinated well water (10 cm water depth) and equipped with some aquatic weeds (*E. densa*) to minimize the stress of examined individuals. Water level was kept exactly at the same height during sound recording to avoid variation in the resonant frequency of tanks used (Akamatsu et al., 2002). Haphazardly caught newts were individually placed in laboratory tanks at least one hour prior to sound recording trials (19:00–22:00). All trials were performed in complete darkness, due to newts being mostly crepuscular to nocturnal. Newt sounds were recorded for 10 min at a 96 kHz sampling rate using a hydrophone (AS-1; Aquarium Scientific, Anacortes, WA, USA; sensitivity = −208 dB re 1V/µPa; linear range = 1 Hz–100 kHz ± 2 dB) connected to a preamplifer (PA-4, Aquarium Scientific), digital-analog converter (ZOOM UAC-2, Zoom North America, Hauppauge, NY, USA) and PC unit with Raven Pro software (v1.4, Cornell Laboratory of Ornithology, Ithaca, NY, USA). The hydrophone was fixed in the middle of the tank 5 cm below the water surface. All other electrical devices were switched-off in the environmental chamber to reduce background noise during recording. After trials, newts were weighed (to 0.01 g) using digital balances (440-33N, Kern, Balingen, Germany).

We analyzed sound recordings using the Raven Pro software. Measurements in this study were selected using spectograms, which had a Hanning window bandwidth of 270 Hz and a frame length of 512 points. Given the newts produced only simple clicks, i.e., short shape sounds, we characterized them using five parameters: 1. duration (ms), difference between the beginning and end of click; 2. pulse interval (s), the time between two consecutive clicks; 3. low frequency (kHz), the lowermost click frequency; 4. high frequency (kHz), the uppermost click frequency; 5. peak frequency (kHz), click frequency with the highest energy. As most individuals produced sounds at short pulse intervals, we measured the first 50 clicks from each newt.

### Statistical analyses

**Figure 2:**
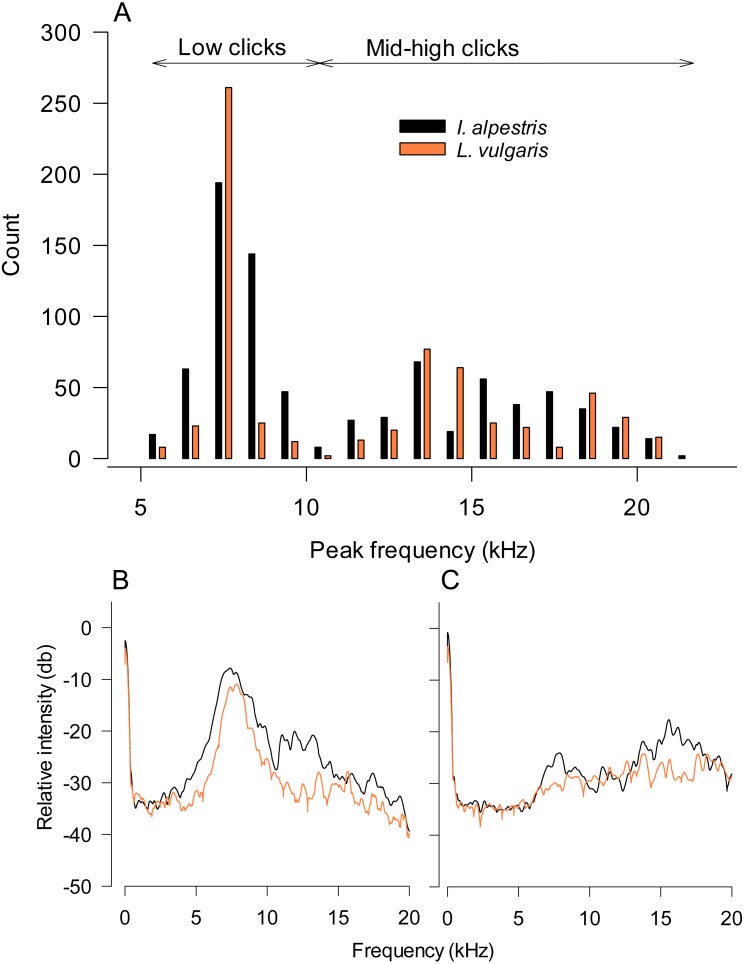
Characteristics of underwater sounds in newts. (A) Peak frequencies of underwater clicks produced by two newt species. Note that both distributions are bimodal with a clear distinction between low and mid-high frequency clicks. (B, C) Power spectra of low (B) and mid-high frequency clicks in both species. Values are averages from all individuals (five clicks per individual). Legend in (A) refers to all graphs.

The distribution of peak frequencies in all measured sounds was clearly bimodal in both species (Fig. 2). Accordingly, we analyzed low- (hereinafter low) and middle- to high-frequency (hereinafter mid-high) clicks separately. To examine individual sound variation, we randomly selected five low and five mid-high clicks from each individual. The number of repeated measurements was chosen relative to the sample size and number of clicks in each category. The effect of individual identity (random factor), sex, species and body mass (covariate) on sound parameters was tested using a permutation general linear model (pGLM). Given the low sample size, the permutation approach (number of permutations = 9,999) was used (Quinn & Keough, 2002). All values are presented as means with 95% CIs. Confidence intervals were calculated using a bias-corrected and accelerated bootstrapping procedure (9,999 bootstrap replications) in the R package “boot” (Canty & Ripley, 2017). Statistical analyses were performed using the Permanova module in Primer (version 6.1.16, PRIMER-E Ltd., Lutton, UK).

## Results

**Figure 3:**
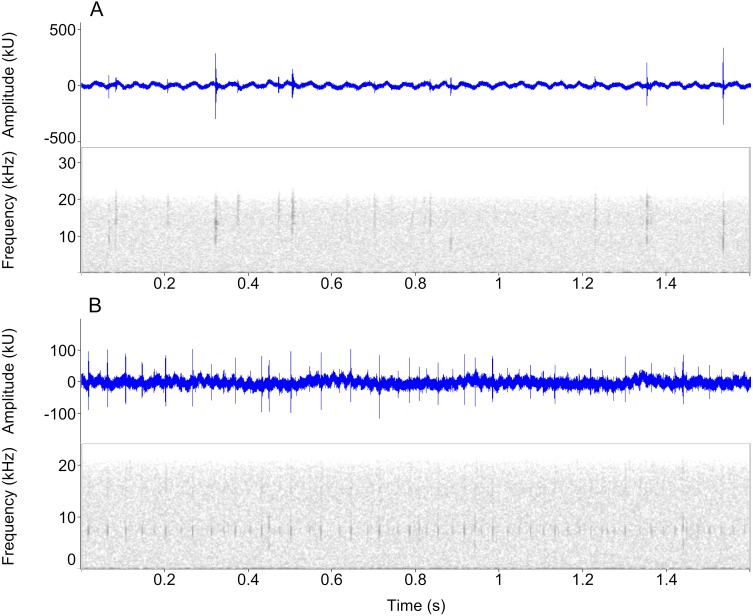
Waveform and spectrogram views of underwater sounds. Waveform and spectrogram views of underwater clicks in two newt species, (A) *Ichthyosaura alpestris*, (B) *Lissotriton vulgaris*. All spectrogram values are from one individual. Note the marked within-individual variation in click frequencies.

In total, we measured 805 clicks from 30 individuals (Fig. 3; Table 1; Audio S1–S4). The required number of clicks (*n* = 50) was obtained from 26 newts, due to marked variation in individual sound production (Table 1). The total sound production did not vary between species (*F*_1,27_ = 1.06, *P* = 0.35) and sex (*F*_1,27_ = 1.25, *P* = 0.30). The proportion of low-clicks ranged from 0 to 100%, but was unaffected by species and sex (species: *F*_1,27_ = 0.18, *P* = 0.68; sex: *F*_1,27_ = 0.07, *P* = 0.78; Fig. S1). Accordingly, we obtained a lower than required number (*n* = 5) of low and mid-high clicks in three and eight newts, respectively.

Species and sex had a negligible effect on sound parameters in both low (Multivariate pGLM: species: *F*_1,26_ = 0.64, *P* = 0.44; sex: *F*_1,26_ = 0.13, *P* = 0.89) and mid-high clicks (species: *F*_1,25_ = 1.35, *P* = 0.27; sex: *F*_1,25_ = 1.39, *P* = 0.24). Sound parameters were affected by individual identity (low clicks: *F*_26,111_ = 4.38, *P* < 0.001; mid-high clicks: *F*_25,97_ = 3.01, *P* < 0.001), which explained 46% and 40% of total variance in low and mid-high clicks, respectively.

**Figure 4:**
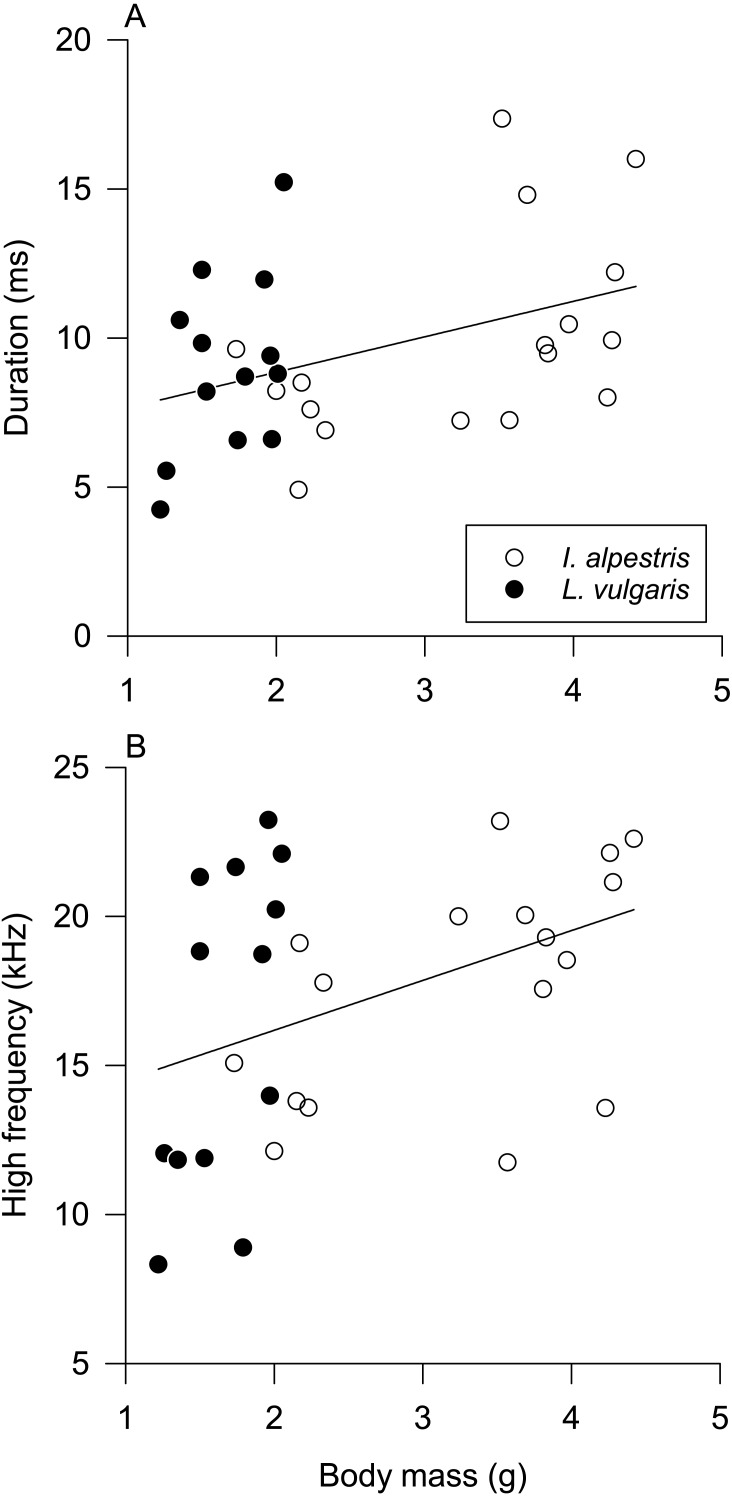
Influence of body mass on sound parameters. Influence of body mass on (A) duration and (B) high frequency of underwater clicks in newts. See Table 2 for statistical results. Data from both species are fitted with ordinary least squares regression for illustrative purposes only.

Univariate pGLMs produced results similar to their multivariate counterparts (Table 2). Species and sex had a statistically nonsignificant effect on all sound parameters (Figs. S2 and S3). The duration and high frequency of low clicks increased with body mass (Fig. 4). All parameters but one were affected by individual identity (Table 2). Individual differences explained 38–53% of total variation in sound parameters, except the peak frequency of low clicks (17%).

**Table 2:** Results of permutation general linear models. Results showing the effect of body mass, species, sex, and individual identity (ID) on parameters of low and middle to high frequency clicks in two species of sympatric newts. Note that *F* values are in fact pseudo *F*-values, because of the unknown *F* distribution in permutation models. Statistically significant values (*α* = 0.05) are in bold.

Parameter	Factor	Low clicks			Mid-high clicks		
		*F*	df	*P*	*F*	df	*P*
Duration							
	Body mass	5.10	1, 26	**0.03**	0.26	1, 29	0.60
	Species	2.13	1, 26	0.15	0.48	1, 25	0.50
	Sex	0.14	1, 26	0.71	0.59	1, 25	0.47
	ID	6.81	26, 113	**<0.001**	5.98	25, 97	**<0.001**
Interval							
	Body mass	0.44	1, 26	0.55	0.32	1, 31	0.58
	Species	2.49	1, 26	0.12	1.01	1, 25	0.34
	Sex	0.24	1, 26	0.63	3.19	1, 25	0.09
	ID	3.01	26, 111	**0.009**	3.84	25, 97	**0.02**
Low frequency							
	Body mass	2.94	1, 26	0.10	1.70	1, 34	0.20
	Species	0.13	1, 26	0.72	3.08	1, 25	0.08
	Sex	0.25	1, 27	0.62	2.59	1, 25	0.12
	ID	2.19	26, 113	**0.008**	2.74	25, 97	**<0.001**
High frequency							
	Body mass	7.19	1, 26	**0.009**	2.34	1, 32	0.14
	Species	0.84	1, 26	0.37	0.02	1, 25	0.89
	Sex	0.05	1, 27	0.82	0.23	1, 25	0.69
	ID	5.47	26, 113	**<0.001**	3.75	25, 97	**<0.001**
Peak frequency							
	Body mass	0.07	1, 26	0.87	1.78	1, 34	0.19
	Species	0.05	1, 26	0.82	0.11	1, 25	0.75
	Sex	1.15	1, 27	0.29	0.84	1, 25	0.37
	ID	1.21	26, 113	0.20	2.72	25, 97	**<0.001**

## Discussion

Although underwater sound production has previously been reported in four families of caudate amphibians, i.e., Ambystomatidae, Proteidae, Salamandridae, and Sirenidae (Gehlbach & Walker, 1970; Wyman & Thrall, 1972; Davis & Brattstrom, 1975; Crovo, Zeyl & Johnston, 2016), our study is the first to analyze species, sex, and individual variation in this trait. Parameters of produced clicks highly overlapped between species and sexes. In contrast, a large amount of variation in underwater sounds was individual-specific.

Our study demonstrates underwater sound production in two salamandrid genera, *Ichthyosaura* and *Lissotriton*. Until now, it has only been reported in the North-American salamandrid genus *Taricha* (Davis & Brattstrom, 1975). According to the well-supported salamandrid phylogeny (Wiens, Sparreboom & Arntzen, 2011), all sound producing lineages split from their common ancestor ca. 60 mya. This suggests that underwater sound production is a shared trait among newts, or sound production evolved independently in at least two or three lineages. In addition, both closely related European taxa produce markedly shorter clicks and at much higher frequencies than *Taricha*, which also concurs with salamandrid phylogeny. However, this comparison should be made with caution because of differences in the equipment used; note the high sample rate and wide linear range of the hydrophone we used in comparison with the previous study. Hence, methodologically consistent data from more taxa are needed for meaningful comparison and ancestral reconstruction of the evolution of underwater sound production within this group.

The most notable aspect of newt underwater sounds is their high peak frequency. Values of mid-high frequency clicks are above known peak frequencies in other salamander taxa (Gehlbach & Walker, 1970; Wyman & Thrall, 1972; Davis & Brattstrom, 1975; Crovo, Zeyl & Johnston, 2016). Specifically, peak frequencies of underwater sounds in European taxa were about 4 kHz higher than in *Amphiuma* and more than 10 kHz higher than in other species. It is questionable as to whether newts hear these high-frequency sounds at all. Acoustic sense abilities are unknown in the examined taxa. Hearing ability in the North-American newt genus *Notophthalmus* is less sensitive than in other salamanders (Zeyl & Johnston, 2016). Generally, salamanders and newts lack a tympanum and middle ear, but they may detect sounds through extratympanic pathways, such as an air-filled mouth cavity (Bulog & Schlegel, 2000) or air volumes in their lungs (Christensen et al., 2015). In addition, recent studies have demonstrated newt phonotaxis using species-specific anuran vocalization cues in several taxa (Diego-Rasilla & Luengo, 2004; Diego-Rasilla & Luengo, 2007; Pupin et al., 2007; Madden & Jehle, 2017), which suggests they are able to detect and discriminate between relatively high frequency sounds. However, the exact range of newt hearing frequencies remains to be determined.

Newt underwater sound production varied among individuals, not between sexes and species. Without further information about the behavioral context of sound production and newt hearing abilities, interpretation of these findings is necessarily speculative. The absence of sex and species differences in sound parameters and sound production in isolation from other individuals suggests that underwater clicks have a limited function in sex recognition or sexual selection. However, two parameters of produced low-frequency clicks, duration and high frequency, were affected by body mass. Although their relationship with body mass is weak, it suggests that clicks may provide some information about the body size of the sound producer in the absence of visual cues. Hence, these clicks have some potential for sex recognition in alpine newts because of their prominent sexual size dimorphism (Colleoni et al., 2014).

Assuming that newts detect individual acoustic differences, sound cues may provide information about the number of individuals in their proximity. This may reduce intra- and interspecific competition in these taxa (Janča & Gvoždík, 2017; Hloušková et al., 2018). In addition, if the sex and species identity of approaching individuals is determined using chemical scents (Malacarne & Vellano, 1987; Cogälniceanu, 1994; Treer et al., 2013), acoustic cues may contribute to this recognition by their combination with olfactory cues in darkness. Simple high-frequency clicks may also be used for echolocation (Gehlbach & Walker, 1970). This explanation assumes high frequency hearing in newts and sufficient intensity of their sounds to produce acoustic reflection from surrounding objects. However, evidence for high frequency hearing is mixed in salamanders and their underwater sounds have quite a low amplitude (see references above). Finally, it is also possible clicking may merely be a byproduct of jaw movements during the detection of olfactory cues under water (Maslin, 1950). Given the marked within- and among-individual variation in pulse intervals and peak frequencies of produced clicks, the later option seems unlikely in both species studied.

In our study, newt underwater sound recordings were performed in relatively small plastic tanks. Although plastic tanks may have disparate acoustic properties compared to glass aquaria, one may argue that sound peak frequencies were affected by the resonant frequency of tanks used (Akamatsu et al., 2002). The calculated resonant frequency of these tanks is 5 kHz. However, note that their real resonant frequency should be somewhat higher, because of the lowered water level. If the resonant frequency indeed affected our sound recordings, it should be visible as peaks in the power spectra of recorded low and high frequency sounds in both species (Figs. 2B and 2C). Although we cannot rule out that the tank resonant frequency somewhat affected the accuracy of peak frequency estimates in low-frequency clicks, parameters of high-frequency sounds seem unaffected by this potentially confounding factor.

## Conclusions

Our study reported remarkable individual, not sex or species, variation in underwater sound production in newts. Information about the magnitude of variation in sound parameters will allow calculation of minimum sample size, which greatly improves the experimental design of future acoustic studies in newts. Our findings also provide exciting new research agendas for further studies on both the causes and consequences of underwater sound production in this group. At a causal level, attention should be dedicated to the mechanisms of sound production and hearing abilities. Understanding the ecological and evolutionary consequences of underwater sound production will require further experimental and comparative studies. In addition, newts respond negatively to artificial sounds (Madden & Jehle, 2017), and so the potential acoustic interaction between newt sound production and anthropogenic noise will be interesting from an applied ecology view. Finally, although both species have been intensively studied for more than 250 years (Roček, Joly & Grossenbacher, 2003; Schmidtler & Franzen, 2004), our results demonstrate that even the natural history of these threatened amphibians is still insufficiently understood.

##  Supplemental Information

10.7717/peerj.6649/supp-1 Figure S1 Proportion of low frequency clicks Individual proportions of low frequency clicks in two newt species. Values are means with 95% CIs.

10.7717/peerj.6649/supp-2 Figure S2 Duration and interval between clicks Duration and interval between low and mid-high frequency clicks in two newt species. Values are means with 95% CIs.

10.7717/peerj.6649/supp-3 Figure S3 Frequency characteristics of clicks Low, high, and peak frequencies of low and mid-high frequency clicks in two newt species. Values are means with 95% CIs.

10.7717/peerj.6649/supp-4 Audio S1 Underwater sounds in *Ichthyosaura alpestris* female

10.7717/peerj.6649/supp-5 Audio S2 Underwater sounds in *Ichthyosaura alpestris* male

10.7717/peerj.6649/supp-6 Audio S3 Underwater sounds in *Lissotriton vulgaris* female

10.7717/peerj.6649/supp-7 Audio S4 Underwater sounds in *Lissotriton vulgaris* male

## Additional Information and Declarations

### Field Study Permissions

The following information was supplied relating to field study approvals (i.e., approving body and any reference numbers): The Agency for Nature Conservation and Landscape Protection of the Czech Republic issued permission to capture the newts (KUJI 224/2013).

### Data Availability

The following information was supplied regarding data availability: Data is available in Figshare: Hubáček, Jiří; Šugerková, Monika; Gvozdik, Lumir (2018): Underwater sounds in newts. figshare. Fileset. https://doi.org/10.6084/m9.figshare.7228370.v1.
