# Supplementary figures and images for: Underwater sound production varies within not between species in sympatric newts
Source: PeerJ. 2019 Mar 28;7:e6649. doi: 10.7717/peerj.6649 (PMC6441559; doi:10.7717/peerj.6649)

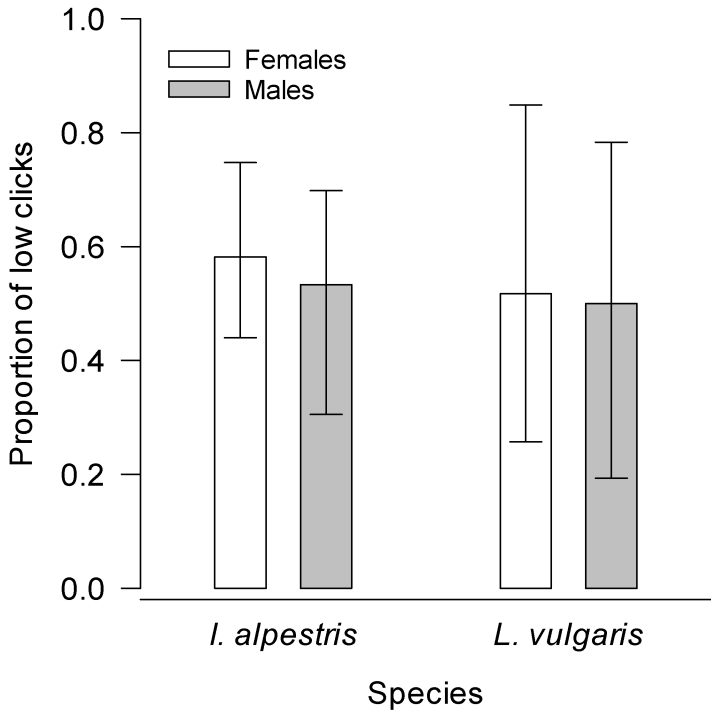

Supplement: Figure S1 — Individual proportions of low frequency clicks in two newt species. Values are means with 95% CIs. [file peerj-07-6649-s001.pdf]

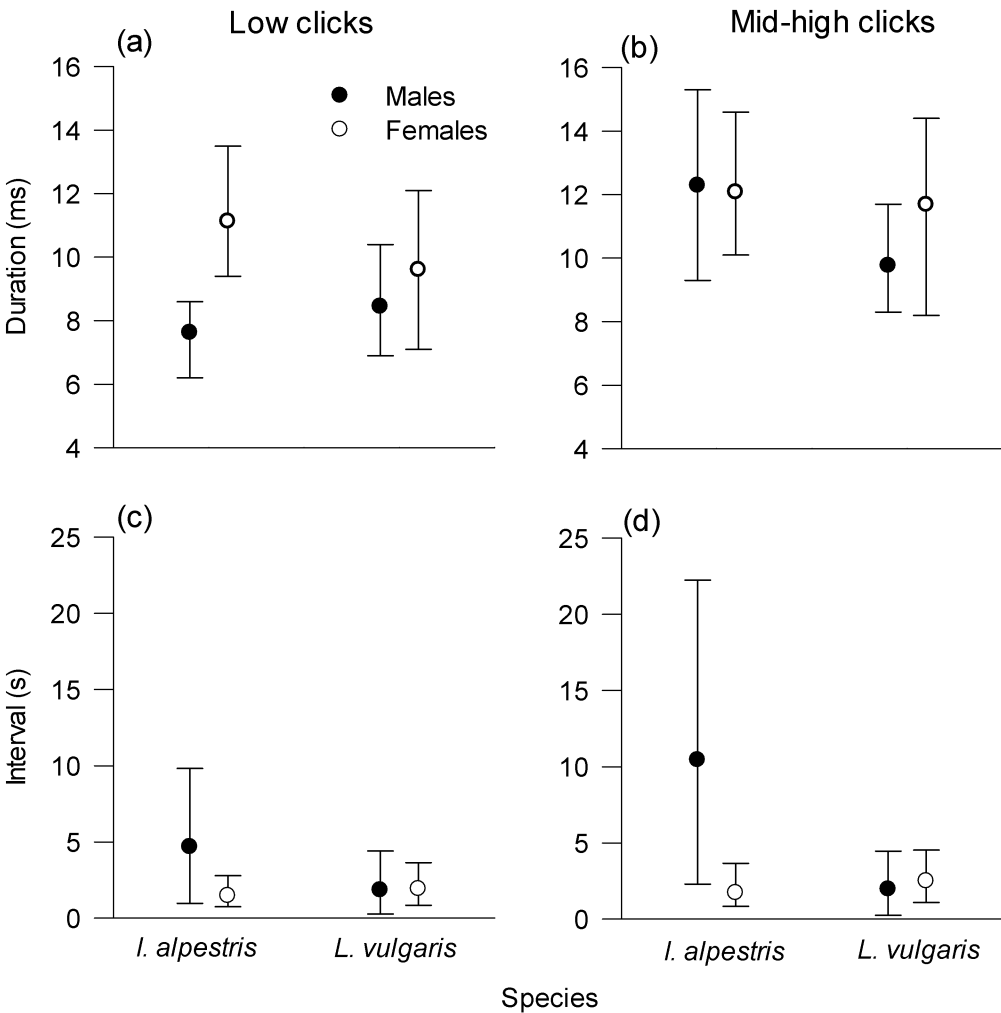

Supplement: Figure S2 — Duration and interval between low and mid-high frequency clicks in two newt species. Values are means with 95% CIs. [file peerj-07-6649-s002.pdf]

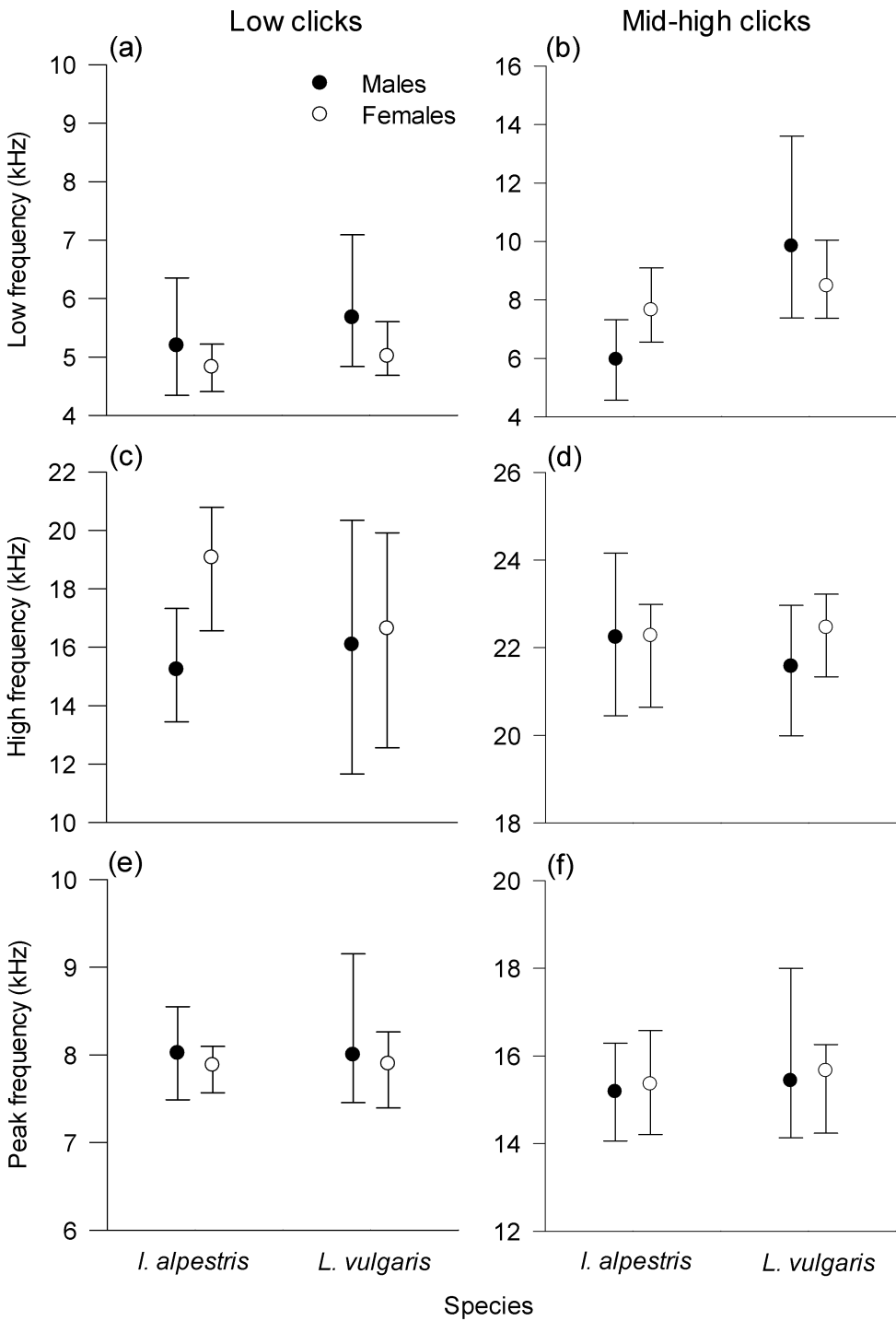

Supplement: Figure S3 — Low, high, and peak frequencies of low and mid-high frequency clicks in two newt species. Values are means with 95% CIs. [file peerj-07-6649-s003.pdf]
